# 

**Published:** 1879-11

**Authors:** 


### BOARD OF EXAMINERS.

The Ohio State Board of Dental Examiners will hold the annual session in Columbus, on Tuesday, December 2nd, at two o'clock, p. m. All especially interested will please be promptly present at that time.

### OHIO STATE DENTAL SOCIETY.

The annual meeting of the Ohio State Dental Society will be held in the city of Columbus, beginning at ten o'clock, a. m., on the first Wednesday of December, prox.

The following subjects are for consideration at that time, viz.:

1. Pathology of the Osseous Structure of the Teeth.

2. Artificial Crowns Engrafted upon the Roots of Natural Teeth.

3. New Modes of Mounting Artificial Teeth.

4. Cases in Practice. Members limited to one case each.

5. Fetor of the Breath; Cause and Treatment.

6. Residual Alveolar Abscess; Cause and Treatment.

7. What is the Best Material for Filling the Roots of Pulpless Teeth?

8. Irregularities of the Teeth.

It is desirable that every member should come up on that occasion prepared to add something of interest and importance. All will go with the desire to learn, and they should also be quite as willing to impart. Every one, by a little timely preparation, can do something in this direction.

It is desirable that all the members be present, also that all the worthy members of the profession in the state become members.

All members of the profession are cordially invited to be present, and add to the interest and importance of the meeting, by their presence at least.

The meeting will continue till Friday afternoon.

### MATRIMONIAL.

Married, September 4, 1879, at the home of the bride's mother, at Milford, O., Mr. E. C. Harding to Miss Katie L. Hendrick, daughter of the late L. A. Hendrick, Jr., M. D., D. D. S.

May the new way upon which this happy couple have just entered, be a bright, joyous and prosperous one, from the beginning to the end.

### CONTENTS.

COMMUNICATIONS.

*THE DENTAL REGISTER,*

A MONTHLY MAGAZINE DEVOTED TO THE INTERESTS OF

THE DENTAL PROFESSION.

*Terms Reduced to $2 50 per Year. Single Copies 25c.*

*EDITED BY PROF. J. TAFT, D. D. S.,*

AND PUBLISHED BY

SPENCER & CROCKER, Ohio Dental Depot.

The volume commences in January and closes in December.

The REGISTER being issued monthly is always fresh, therefore Indispensable to the practitioner who desires to keep posted up with the new inventions and improvements which are daily developed. Neither expense nor effort will be spared to give value and variety to its contents. Articles will be illustrated with well executed engravings whenever they will add to the interest or assist to explanation.

Its extensive circulation and frequency of issue make it one of the BEST DENTAL ADVERTISING MEDIUMS in the United States.

All subscrptions must begin with January or July.

Local or special notices, five lines of less, will be inserted for $3.00 each insertion; over five lines, 50 cts. per line.

All advertising bills payable in advance, or at furthest, after the issue of the first number containing the advertisement. All transient advertisements to be paid for in advance.

☞CONTRIBUTIONS SOLICITED.

Exclusively Editorial Communications should be addressed to Editor.

The latest number of the REGISTER may be ordered with or without other goods at any time, and all letters should be addressed to

**SPENCER & CROCKER, Ohio Dental Depot, Cincinnati, O.**

